# Bone defect repair materials after bone tumour resection: from structural substitutes to biofunctionalized synergistic therapy

**DOI:** 10.1080/07853890.2026.2700085

**Published:** 2026-07-25

**Authors:** Hongyu Ou, Fei Liu, Wei Wang

**Affiliations:** Department of Orthopaedic Oncology, Liaoning Cancer Hospital & Institute, Shenyang, China

**Keywords:** Bone tumour resection, bone defect repair, biofunctional scaffold, Intelligent drug delivery, additive manufacturing, angiogenesis–osteogenesis

## Abstract

**Background:**

Critical-sized bone defects following malignant tumour resection remain among the most difficult challenges in orthopaedic oncology. Conventional substitutes such as autografts or polymethyl methacrylate (PMMA) provide initial stability but often fail to ensure durable integration or prevent recurrence. This review aims to summarize recent progress in bone defect reconstruction materials, with particular attention to strategies that unite structural reliability with biological functionality.

**Discussion:**

Over the past decade, repair materials have evolved from inert fillers to multifunctional scaffolds capable of combining load-bearing support with local therapeutic action. Current designs integrate chemotherapy, photothermal hyperthermia or magnetic hyperthermia, immunomodulation, antibacterial function, and angiogenic–osteogenic cues. Intelligent drug delivery systems and additive manufacturing enable patient-specific, stimuli-responsive implants, while early clinical use of antibiotic-loaded cements and custom-printed prostheses demonstrates translational potential. Nevertheless, most evidence remains preclinical and heterogeneous, with inconsistent tumour, infection, regeneration, and safety endpoints; long-term safety, manufacturing reproducibility, cost-effectiveness, and regulatory acceptance remain unresolved.

**Conclusions:**

Reconstruction after bone tumour resection is entering a new era in which implants are expected not only to restore anatomy and stability but also to actively suppress tumour recurrence and foster regeneration. Multifunctional and patient-tailored scaffolds hold promise to redefine limb-salvage surgery, provided that future work delivers robust clinical validation and scalable manufacturing pathways.

## Introduction

Reconstructing critical-sized bone defects following tumour resection remains clinically challenging [1–3]. While autografts and porous PMMA offer mechanical support, their limited bioactivity frequently results in tumour recurrence and inadequate osseointegration [4]. Emerging biomaterials address this by combining structural functionality with bioactive properties, enabling synchronized degradation and osteogenesis while preserving mechanical integrity [5]. These advanced systems incorporate therapeutic mechanisms such as controlled drug release, immunomodulation, and photothermal ablation [6]. Tumour microenvironment-responsive platforms have been reported to enable precise spatiotemporal control over drug delivery while simultaneously supporting coupled osteogenesis and angiogenesis. Such innovations transform traditional scaffolds into dynamic therapeutic constructs.

**Figure 1.**
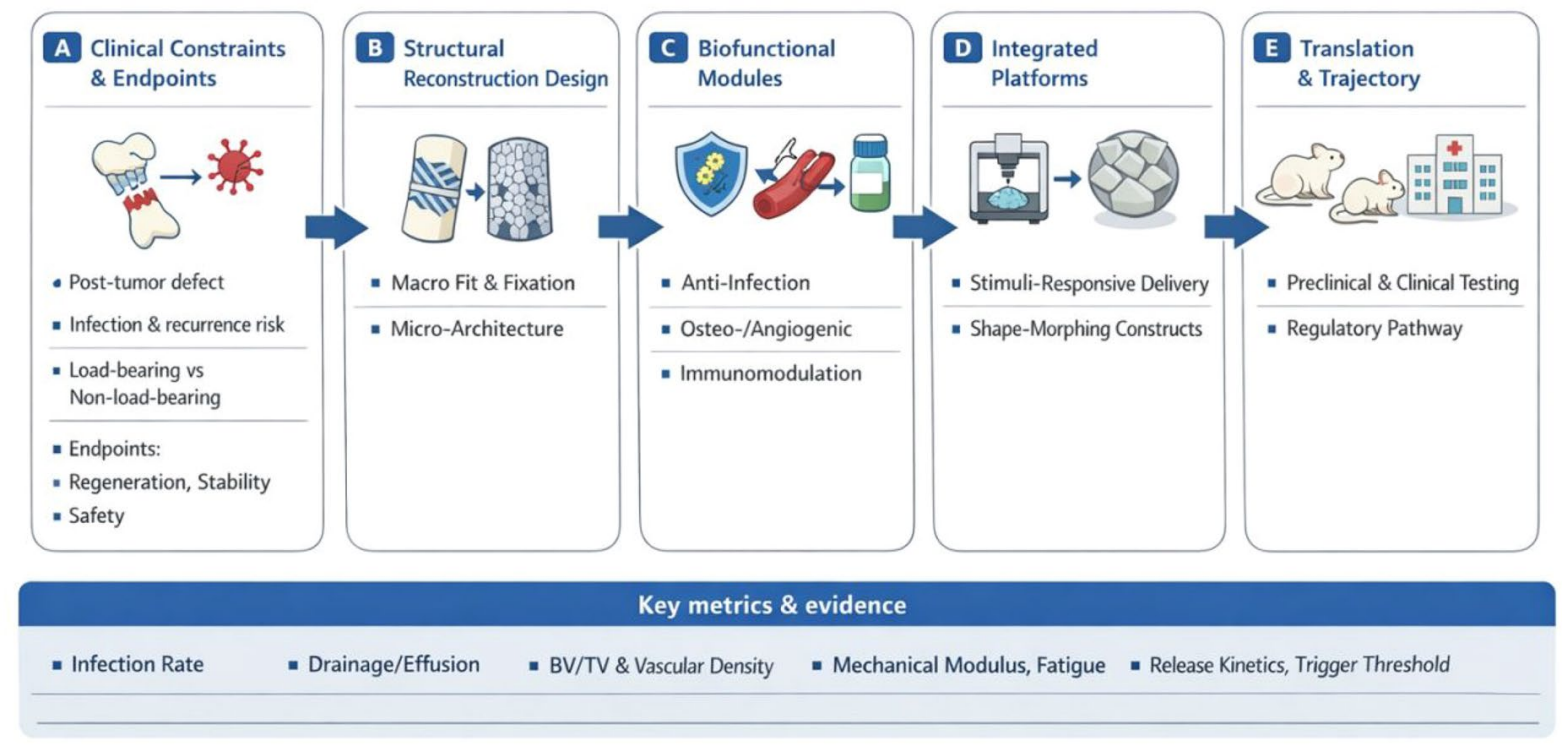
Roadmap.

This review examines these dual-function materials and establishes a conceptual framework for integrated “repair–therapy” approaches. In post-tumour bone defects, an optimal repair strategy must simultaneously address load-bearing structural reconstruction, local tumour control, infection prevention, vascularized bone regeneration, and translational feasibility. However, most available evidence remains preclinical and heterogeneous. Many studies rely on *in vitro* assays, ectopic implantation, or small-animal defect models that do not fully reproduce the clinical environment after tumour resection, including residual tumour burden, chemotherapy- or radiotherapy-related impairment, compromised soft-tissue coverage, infection risk, and complex load-bearing conditions. Moreover, endpoints vary substantially across studies, including tumour volume, apoptosis, bacterial burden, micro-CT indices, histology, vascular markers, and mechanical testing. Therefore, this review not only summarizes material design strategies but also evaluates their translational maturity, model limitations, and implementation barriers (Figure 1. Roadmap).

## Limitations of alternative materials for traditional structures

Post-tumour bone defects impose stringent structural reconstruction requirements, and achieving durable mechanical stability remains a primary bottleneck in limb-salvage repair. In this section, we examine the structural design dimension by clarifying why conventional reconstructive substitutes fall short. We also summarize how key failure mechanisms—mechanical mismatch, inadequate osseointegration, and degradation–osteogenesis asynchrony—emerge across traditional material systems. These limitations motivate the subsequent discussion of biofunctionalized therapeutic strategies that can be integrated with structural constructs to regulate the local microenvironment and improve long-term outcomes.

Over the past decades, structural replacement has remained the cornerstone of limb-salvage treatment after bone tumour resection. Metal plates, intramedullary nails, bone cement, and biological grafts restore continuity and enable early function, but long-term follow-up consistently shows that conventional substitutes cannot fully meet the demands of segmental reconstruction. Failures largely reflect three interrelated challenges: mechanical mismatch, unreliable osseointegration, and degradation–osteogenesis asynchrony.

Mechanical mismatch remains the first obstacle. Substitutes must restore axial and torsional stability without overstressing adjacent bone, yet conventional fixation rarely replicates the torsional resistance and fatigue endurance of native bone [7–10]. Long-term studies confirm that autografts, allografts, and cement each fail in different ways—fracture, loosening, or degeneration [11–13]. Patient-specific 3D-printed lattices can distribute load more evenly [14], but their long-term strength and durability, especially with resorbable composites, remain uncertain [15].

Biological integration is equally critical. An inert interface may provide early support but undermines osseointegration and increases infection risk. Cement is mechanically reliable but biologically passive, with mid-term reports of cartilage wear and interface breakdown [16–19]. Resorbable substitutes such as β-TCP/PLGA or collagen composites encourage bone ingrowth yet show unpredictable remodelling [20–23]. Custom titanium lattices and bioactive glass offer promise, but most reports are short-term or limited to low-load sites [24–27].

Degradation–osteogenesis asynchrony compounds both problems. Ceramics or polymers that resorb too fast may collapse before bone heals, while slow-resorbing HA or persistent collagen can block remodelling and complicate imaging [28–31]. Composite systems attempt to balance this mismatch by coupling degradable phases with osteogenic cues [32], but evidence is limited to short follow-up. The host environment—vascularity, tumour biology, and defect size—further shifts the balance, underscoring the need for multiparameter design [33].

These limitations explain why complications remain frequent in current reconstructions and why conventional substitutes fall short in durability and biological engagement. They provide the rationale for the transition from passive reconstruction toward functionalized, bioactive implants. Biological reconstructions such as liquid-nitrogen–treated grafts can achieve satisfactory union in selected cases [34], but long-term series also reveal late failures from infection, nonunion, or resorption [11,35]. Even surgical techniques influence outcomes; for example, high-speed burring may aerosolise residual tumour particles, raising concern for local seeding [36,37].

Collectively, these observations highlight that anatomical compatibility or temporary stability alone cannot ensure lasting success. Durable reconstruction requires implants that combine mechanical reliability with active biological participation. This paradigm shift sets the stage for the biofunctional strategies discussed in the next section.

### Antitumour functionality

While traditional structural substitutes can restore continuity, their long-term performance remains constrained by incomplete biological control of the post-resection microenvironment, particularly with respect to local disease eradication. In this section, we shift to the ‘therapy’ dimension of repair–therapy integration by summarizing antitumour functionalities enabled by local delivery platforms, including drug-eluting scaffolds and mechanism-guided strategies designed to suppress residual tumour while preserving osteogenic potential. These approaches also underscore the broader need for multifunctional bioactive systems—such as anti-infective and immunomodulatory modules—that will be discussed in the next section.

High recurrence rates after bone tumour resection highlight the need for implants that provide both structural repair and local tumour suppression [38–40]. A common theme across modalities is that local delivery achieves high drug concentration at the defect site with reduced systemic toxicity, while many systems simultaneously promote osteogenesis or angiogenesis. The following strategies illustrate how different mechanisms have been applied within this framework.

Localized chemotherapy most commonly employs drug-eluting scaffolds. Polymers, bioactive ceramics, and titanium frameworks enable the sustained local release of agents like cisplatin or doxorubicin, which inhibit tumour growth while preserving osteogenic potential [41–47]. Smart carriers, including pH-responsive designs, further improve selectivity in acidic niches [48,49]. Bisphosphonate-based systems add the advantage of inhibiting osteoclasts while supporting bone [50–52]. Where reported, tumour control is typically quantified by tumour growth/weight or apoptosis markers, while repair compatibility is assessed by micro-CT/histology (e.g. BV/TV, new bone area) in small-animal models; however, evidence remains limited to *in vitro* and small-animal studies, with no published human trials.

Photothermal and magnetic hyperthermia (MHT) therapies have been integrated into scaffolds using nanomaterials such as polydopamine (PDA) or MXene to enable local tumour ablation while supporting regeneration [53–63]. Representative MXene–bioactive glass and Mn_3_O_4_-enhanced hydrogel systems have achieved local photothermal or magnetothermal tumour suppression while supporting bone formation in preclinical models [59,64–67]. However, efficacy depends strongly on local heat dissipation, treatment conditions, and temperature–tissue safety balance. Clinical translation will require safe energy delivery, standardized thermal dosimetry, and paired reporting of tumour endpoints, such as recurrence or tumour growth curves, alongside repair outcomes, such as BV/TV and mechanical restoration.

Gene delivery strategies employing scaffolds remain less common. Nanoparticles loaded with siRNA and gene-activated matrices encoding cytokines or BMPs demonstrate tumour inhibition alongside osteogenic support *in vitro* and in small-animal models [51,68], but instability, limited transfection efficiency, and regulatory barriers constrain near-term use. Immunomodulatory strategies have advanced with the development of scaffolds that release CSF-1R inhibitors, checkpoint blockers, or cytokines to reprogram macrophages and T cells for tumouricidal activity while simultaneously supporting bone repair [69,70]. These align with oncology trends but face challenges in protein stability, inflammation control, and regulatory approval.

Most single-modality approaches remain preclinical, mainly in rodent models. Drug-loaded cements and ceramics are closest to clinical use, but tumour-specific applications await validation.

Scaffolds are increasingly designed to integrate multiple therapeutic modalities, aiming for synergistic tumour suppression and regeneration. The most common model combines chemotherapy with photothermal therapy, where composites such as PDA frameworks or iron-containing scaffolds achieve drug release and localized heating, enhancing cytotoxicity while promoting osteogenesis [64,71,72]. Extensions include chemodynamic therapy, in which iron-based components catalyze Fenton/Fenton-like reactions to generate ROS and deepen tumour killing while retaining osteogenic cues [73–75]; in parallel, kill-then-regenerate sequencing is increasingly implemented by decoupling early ablative conditions from later pro-osteogenic conditions—e.g. the Mn_3_O_4_ hydrogel above explicitly separates a 43 °C ablation window from *a* ∼40 °C osteogenic window [67], and intelligently sequential scaffold designs have been reported that switch from ROS-amplified tumour apoptosis to anti-inflammatory, pro-osteogenic microenvironment remodelling after implantation [76]. Sequential release systems further refine control by providing an early cytotoxic burst followed by sustained osteogenic cues [77–79].

The rationale is clear: single therapies may leave resistant cell populations, whereas dual or triple strategies broaden tumour eradication and may improve regenerative outcomes. However, these benefits remain supported mainly by preclinical data. Key barriers include tissue safety, reproducibility of printed or composite scaffolds, long-term biocompatibility of novel nanomaterials, and access to standardized laser, ultrasound, or magnetic field systems [80,81].

Closer-to-practice approaches include drug-eluting cements and ceramics, which are already familiar in load-bearing contexts [82–87]. Magnetic nanocomposite cements have controlled tumours in rabbit models, suggesting feasibility for larger sites [64]. Immunomodulatory scaffolds remain at an earlier stage but may complement systemic cancer therapies. Clinical application will likely depend on tumour biology: aggressive lesions may require immediate ablative or cytotoxic designs, whereas low-grade disease could benefit from osteo-promoting implants such as zoledronate (ZOL)-releasing scaffolds. In the longer term, modular platforms capable of incorporating interchangeable ‘cartridges’ may provide the greatest adaptability.

Implantable multifunctional scaffolds represent a shift from passive reconstruction to biologically active therapy. Proof-of-concept studies support local tumour control with concurrent bone regeneration, but current evidence remains confined to rodent and occasional large-animal studies, with no published human trials. Translation will require precise release control, biomechanical reliability, long-term safety validation, manufacturing reproducibility, and clearer regulatory pathways [88].

### Enhanced antibacterial function

Postoperative infection remains a major threat in limb-salvage surgery for malignant bone tumours, where treating infection and repairing large defects must proceed in parallel [89–92]. To address this, researchers are actively developing repair materials with intrinsic or integrated antibacterial properties [93]. These strategies broadly fall into antibiotic-based implants, antibiotic-free antiseptic doping, and multifunctional composites or nanomaterials, all aiming to prevent infection without impairing bone regeneration.

Antibiotic-loaded scaffolds have been widely applied to deliver high local drug concentrations [94–98]. A notable example is the 3D-printed spacer customized for a paediatric osteosarcoma case, which allowed precise placement of antibiotic bone cement and resulted in infection eradication and limb salvage [89]; in a comparable two-stage periarticular infection case, a personalized 3D-printed mould–assisted antibiotic PMMA spacer eradicated MRSA and remained stable at 2-year follow-up [99]. While effective, this approach is limited by finite release and resistance concerns, prompting exploration of antibiotic-free alternatives.

Antiseptic doping offers one such path. Iodine-infused calcium phosphate cement sustained bactericidal activity against *S. aureus* and *E. coli* for up to eight weeks while preserving osteoconductivity, highlighting that broad-spectrum protection can be achieved without antibiotics [100]; a rabbit femoral implantation study similarly reported maintained cytocompatibility and bone formation around 5% iodine CPC at 8 weeks, alongside sustained antibacterial activity *in vitro* [100].

Building further, multifunctional platforms seek to unify antibacterial protection with oncologic control and regeneration. Drug-eluting cements incorporating photothermal/magnetothermal and antibacterial elements, chitosan/nano-HA scaffolds with zoledronate, and LDH-derived nanosheet–functionalized bioactive glass scaffolds have been reported to combine infection control with tumour suppression or bone repair [101–111]. However, comparability remains limited because bacterial burden, *in vivo* infection recurrence, local cytotoxicity, osseointegration, and bone-volume metrics are not consistently co-reported.

Taken together, these approaches reflect a shift toward integrated antibacterial biomaterials. Antibiotic-loaded PMMA spacers remain the most clinically mature strategy, whereas ion-doped, photothermal, and multifunctional antibacterial scaffolds are still largely preclinical. Clinical translation will depend on standardized reporting of bacterial burden, infection recurrence, local cytotoxicity, osseointegration, antibiotic-resistance risk, long-term safety, and regulatory approval [89,100–102,109].

### Bone regeneration and angiogenesis synergy

Successful repair of tumour resection defects requires synchronized bone formation and vascularization [112–114]. Bone is a highly vascular tissue, and insufficient revascularization results in hypoxia, stalled osteogenesis, and eventual graft failure. Early vascularization removes waste and supplies oxygen, minerals, and growth factors, supporting new bone growth [115,116]. Insufficient angiogenesis remains a key limitation in large defect healing, leading to strategies that combine osteogenic and angiogenic cues to achieve functional repair after tumour surgery.

Multiple studies show that bone morphogenetic proteins (BMPs) and vascular endothelial growth factor (VEGF) can synergistically promote vascularized bone regeneration [117–120]. Scaffold systems such as hydrogels or microspheres provide sequential VEGF–BMP release, ensuring early vessel ingrowth followed by osteoblast differentiation [121,122]. Most evidence remains preclinical (mainly small-animal defects), typically reporting micro-CT and histology for bone (e.g. BV/TV, BMD) alongside vascular readouts (e.g. CD31 staining or perfusion-based angiography), while clinical translation requires optimized dosing to avoid ectopic ossification or aberrant vessels [123,124].

Mesenchymal stem/stromal cells (MSCs) serve as both osteogenic precursors and pro-angiogenic mediators through secretion of VEGF and PDGF [125–128]. Experimental work shows that MSCs accelerate defect bridging, enhance vascular ingrowth, and modulate immune responses without increasing recurrence risk [129–133]. Clinically, autologous bone marrow aspirate concentrates (BMAC) and platelet-rich plasma (PRP) have already been applied in tumour cavities with encouraging early outcomes [41,134]. MSC-seeded bioceramic scaffolds further enhance repair, though safety monitoring is still essential in oncology settings [135].

Implant surfaces can be engineered to create angiogenic niches without exogenous factors. Incorporation of metal ions such as cobalt or copper, or bioactive coatings like polydopamine, has been shown to enhance osteoblast adhesion and upregulate VEGF expression. For instance, modified calcium phosphate or hydroxyapatite scaffolds have promoted both angiogenesis and osteogenesis *in vivo*, while clinical use of bioactive glass demonstrates that surface-driven vascular cues may be readily translated [25,135–138].

The post-resection cavity often contains residual tumour cells, inflammatory mediators, and oxidative stress. Several studies demonstrate that scaffolds delivering macrophage-targeted inhibitors or antioxidant nanomaterials can reprogram the local environment, suppress immunosuppressive infiltration, and support coupled bone–vessel regeneration [69,139,140]. These designs highlight the need to balance anti-tumour efficacy with preservation of pro-healing angiogenesis.

Scaffolds responsive to light, pH, or other stimuli enable spatially and temporally controlled therapy while preserving vascularized repair [141–143]. As a representative NIR-responsive MXene platform, an Nb_2_C MXene-functionalized 3D-printed scaffold enabled *in situ* tumour photothermal ablation (surface temperature ∼56 °C within 3 min vs ∼40 °C in controls) and prolonged survival (47 ± 4.5 days vs ∼20–24 days in controls), while also driving denser peri-implant vascular networks at 3 weeks and higher micro-CT bone indices at later time points [54]. In parallel, sustained-release constructs can support long-window local control: a mesoporous-silica (SBA15NH_2_)–zoledronate system achieved a loading of 236.53 mg/g and release for >6 weeks, inhibiting osteosarcoma proliferation and osteoclast-mediated resorption (TRAP/F-actin ring and pit assays), thereby helping maintain a pro-regenerative bone microenvironment [50]. Janus core–shell hydrogels have also been proposed to program stage-adapted cues (e.g. early tumour suppression and later osteogenic support), though quantitative vascular–bone coupling endpoints should be more consistently reported [77].

Overall, converging evidence supports the principle that coupling angiogenesis with osteogenesis is essential for reliable bone regeneration after tumour resection. Growth factors, stem cells, surface modification, immune modulation, and smart release systems provide complementary routes to vascularized repair. However, excessive VEGF/BMP activity may cause ectopic ossification, aberrant vascularization, or unpredictable tissue formation, and pro-regenerative cues should be evaluated for their potential effects on residual tumour cells. Large-animal, load-bearing models with long-term follow-up are needed to define a safe therapeutic window.

### Immunomodulation of the local microenvironment

Post-surgical bone tumour defects are embedded within a dysregulated immune microenvironment shaped by both tumour biology and surgical trauma. If unchecked, this environment can hinder repair and facilitate recurrence. Excessive M2-polarized tumour-associated macrophages (TAMs) generate an immunosuppressive niche, while unrestrained inflammation can exacerbate tissue damage [69,144,145]. Effective reconstruction, therefore, requires an immune milieu that eliminates residual tumour cells while guiding regeneration. Current strategies include macrophage polarisation regulation, cytokine and chemokine delivery, immune checkpoint modulation, and approaches that exploit immune–osteogenic coupling.

Macrophage polarisation regulation has emerged as a central strategy. Several studies demonstrate that inhibiting CSF-1/CSF-1R signalling with locally delivered antagonists reprograms TAMs away from a pro-tumoural phenotype while simultaneously supporting late-phase bone repair [69]. Likewise, mineral-based scaffolds such as HA-modified or graphene-HA composites have shown intrinsic immunomodulatory effects, tempering post-ablation inflammation and remodelling the microenvironment in ways that both suppress tumour recurrence and promote osteogenesis [144,146,147]. Most evidence remains preclinical; where quantified, endpoints typically include M1/M2 markers (e.g. iNOS/CD86 vs CD206/Arg1), inflammatory cytokines (TNF-α/IL-10), and paired repair readouts (micro-CT/histology for bone formation).

Cytokine- and chemokine-based therapies focus on reshaping the defect niche through multifactorial signalling. Instead of single-factor release systems, biologically derived cocktails such as autologous bone marrow aspirate concentrate (BMAC) with platelet-rich plasma (PRP) create a broad immunomodulatory and osteogenic milieu. This combination provides a surge of anti-inflammatory and pro-healing signals while supporting robust angiogenesis and osteogenesis, and has already demonstrated efficacy in clinical cases of mandibular tumour defects [148]. Compared with engineered scaffolds releasing one or two cytokines, this patient-derived strategy offers a holistic, translationally feasible option; however, its variability and lack of tumour specificity remain limitations. Reporting should ideally include inflammatory control alongside radiographic union and functional outcomes.

Immune checkpoint modulation is another promising avenue when confined to the surgical bed. A perioperative local-release platform delivering checkpoint blockade to the resection cavity improved antitumour immune surveillance (including increased effector T-cell activity), prevented local recurrence, and reduced distant metastases versus systemic/free-drug controls, supporting the rationale for localized checkpoint blockade to reduce systemic exposure while sustaining immune pressure against relapse [149]. In a separate local anti–PD-1 hydrogel strategy, disease progression was suppressed and T-cell surveillance enhanced versus controls in an oral carcinogenesis model [150]. Key translational constraints remain the dose–toxicity window, the risk of excessive inflammation at a healing interface, and the need to co-report tumour endpoints (recurrence/metastasis) and repair endpoints (bone regeneration) within the same study.

Underlying all these strategies is the principle of immune–osteogenic coupling. Macrophages, T cells, and mesenchymal stem cells (MSCs) interact to determine whether inflammation transitions into bone formation or stalls into fibrosis [129,151,152]. MSC-based implants, cytokine-rich biological therapies, and staged-release scaffolds exemplify approaches that synchronize anti-tumour immunity with regenerative processes. Experimental and early clinical evidence support their dual capacity to suppress recurrence and accelerate repair [69,144,148]. Importantly, some materials—such as PMMA–Fe_3_O_4_ composites—have demonstrated immunological neutrality while providing tumour ablation and structural support, emphasizing the importance of safety alongside efficacy [64].

In summary, immunomodulation reframes post-tumour bone repair as a bioimmune challenge rather than a purely structural problem. By tuning macrophage responses, manipulating cytokine milieus, applying local checkpoint inhibitors, or leveraging immune–bone crosstalk, next-generation implants may act as both scaffolds and local immune regulators. A key unresolved issue is the balance between antitumour immune activation and regenerative inflammation: excessive immune activation may impair osseointegration, whereas excessive M2-like polarization may theoretically favour immune escape. Future studies, therefore, require time-resolved immune profiling, long-term follow-up, and harmonized safety readouts rather than simplified M1/M2 endpoint assessment [64,69,129,144,148].

### Intelligent responsive and targeted drug delivery systems

Although emerging biofunctional fillers introduce therapeutic activity, their clinical impact is often limited by non-ideal dosing profiles and a mismatch between treatment timing and the evolving post-resection microenvironment. In this section, we extend the ‘therapy’ axis of repair–therapy integration by summarizing intelligent responsive and targeted drug delivery systems embedded within structural scaffolds, cements, or hydrogels, with emphasis on microenvironment-triggered release and mechanism-guided selectivity. This discussion also motivates the subsequent focus on translational and manufacturing considerations needed to realize reliable, clinically deployable multifunctional implants.

Local recurrence and metastasis remain major concerns after bone tumour resection, as systemic chemotherapy seldom achieves therapeutic drug concentrations at the surgical site and causes significant toxicity [153,154]. Intelligent drug delivery systems aim to overcome this limitation by integrating stimuli-responsive release and targeted delivery into structural scaffolds, cements, or hydrogels [155]. These multifunctional platforms not only reconstruct skeletal defects but also suppress tumour regrowth and support regeneration [51]. Early studies with gelatine-based carriers delivering chemotherapeutics demonstrated improved tumour control while preserving mechanical stability [156], underscoring the transition from inert grafts to bioactive, synergistic implants.

Microenvironment-responsive release leverages the acidic and enzyme-rich milieu of osteosarcoma sites. pH-sensitive titanium scaffolds loaded with paclitaxel prodrugs released their payload only under acidic conditions, achieving tumour clearance *in vivo* [43,157]. Tumour endpoints typically include tumour growth curves/bioluminescence and histology, while repair compatibility requires paired osseointegration readouts such as micro-CT bone indices; these are not consistently reported across studies. Similarly, chitosan/MOF composites (CS/DOX@Ti-MOF) responded to tumour acidity, eradicating residual cells while the MOF backbone promoted osteoconduction [158–160]. Comparators should include free drug and non-responsive scaffolds to separate trigger effects from carrier effects. Sustained-release carriers, such as mesoporous PLLA composites delivering zoledronate, prolonged local therapy for over six weeks and reduced osteoclast-mediated bone loss [50,161,162]. These systems highlight the potential of endogenous cues for selective treatment, though variability in tumour acidity or enzyme levels raises concerns for clinical reliability.

4D printing extends 3D printing by adding time-dependent shape memory or functional evolution (‘program–stimulate’) [163,164]. By enabling minimally invasive deployment and adaptive conformation, 4D implants may reduce dead space and micromotion, while also supporting timed antibacterial or osteogenic functions [163–165]. However, deformation-coupled release kinetics, long-term mechanical durability, sterilisation stability, and quality-control metrics remain insufficiently quantified [164,166,167]. Thus, 4D scaffolds provide a promising spatiotemporal platform for repair–therapy integration but remain at an early translational stage.

Magnetic field-responsive platforms address this depth limitation. Superparamagnetic iron oxide nanoparticles (IONPs) embedded in scaffolds can generate local hyperthermia under alternating fields and provide MRI visibility for image-guided therapy [168]. Magnetic mesoporous calcium–silicate scaffolds with DOX achieved synergistic chemo-hyperthermia while stimulating BMP-2/Smad/Runx2 osteogenic pathways [101,169]. Beyond thermal effects, magneto-mechanical stimulation and drug concentration *via* external fields expand their potential, though translation will require specialized equipment and precise safety calibration.

Enzyme- and chemically responsive systems offer another route, using tumour-associated enzymes to degrade coatings or activate prodrugs. Nanozymes that catalytically generate cytotoxic radicals from tumour metabolites are under exploration [101]. Although still preclinical, such approaches may provide highly selective tumour targeting within bone defects.

A further innovation is programmed or staged therapy, designed to match clinical needs from early tumour clearance to later bone repair. In one ‘self-programmed’ design, a Janus-inspired core–shell hydrogel achieved differential melatonin release intended to suppress residual tumour cells early and support osteogenesis later. *In vivo* antitumour and osteogenic benefits were reported, but key quantitative pairs (e.g. tumour volume/recurrence alongside BV/TV or push-out strength) should be explicitly co-reported for cross-study comparability [77].

More generally, sequential “kill-then-regenerate” platforms aim to couple an early cytotoxic window (chemo/ROS/hyperthermia) with a later pro-osteogenic milieu (osteoconductive phases or osteogenic cues) [170]. A representative sequential scaffold system demonstrated controllable tumour killing followed by defect regeneration, and reported improved bone micro-CT parameters such as BV/TV (e.g. 43.6% vs 28.7% at week 12 in a cranial defect model) and trabecular number (0.40 vs 0.31 mm^−1^ at week 12), illustrating how staged designs can be evaluated using paired tumour and repair endpoints [76]. These systems embody the concept of dynamic, context-adapted therapy, though reliable large-scale manufacturing and precise control of release kinetics remain major challenges.

In summary, intelligent drug delivery systems transform bone tumour reconstruction from a static replacement into an active therapeutic platform. By responding to microenvironmental cues, external stimuli, or programmed release sequences, they integrate tumour suppression with regeneration in a single scaffold. However, tumour acidity, enzyme expression, oxygenation, vascularity, and immune status vary among patients and tumour types, making release behaviour difficult to predict from simplified experimental settings. Clinically relevant validation should therefore test release kinetics across broader microenvironmental ranges and include matched non-responsive controls, free-drug controls, paired tumour and repair endpoints, and long-term safety assessment.

Post-tumour bone reconstruction has moved beyond mere structural replacement. Functions such as tumour inhibition, antibacterial activity, osteogenesis–angiogenesis promotion, immune modulation, and smart drug delivery are increasingly integrated into single platforms. To provide a more integrated comparison rather than a study-by-study description, the major biofunctional scaffold strategies are summarized according to their mechanisms, advantages, limitations, and translational maturity in Table 2.

**Table 2.** Comparative overview of biofunctional scaffold strategies for bone defect repair after bone tumour resection.

Scaffold strategy	Main mechanism of action	Representative platforms	Main advantages	Main limitations	Current translational stage
Drug-eluting scaffolds and cements	Sustained local release of antitumour agents, antibiotics, bisphosphonates, or osteogenic drugs within the defect site	PMMA cement, calcium phosphate cement, bioactive ceramics, polymeric scaffolds, titanium-based carriers [41–52,82–87]	High local drug concentration; reduced systemic exposure; relatively compatible with existing surgical workflows	Burst release; limited control of long-term kinetics; possible local cytotoxicity; uncertain tumour-specific benefit in humans	Closest to clinical use among biofunctional strategies, especially for antibiotic-loaded or drug-loaded cement systems; tumour-specific applications still need validation
Photothermal and magnetic hyperthermia scaffolds	Local tumour ablation induced by near-infrared irradiation or alternating magnetic fields, often combined with osteoconductive scaffold matrices	PDA-, MXene-, Fe₃O₄-, or magnetic nanoparticle-functionalized ceramics, hydrogels, and composite scaffolds [53–67]	Spatially controlled tumour suppression; potential combination with osteogenesis and antibacterial effects	Thermal injury risk; limited penetration for NIR systems; need for standardized thermal dosimetry and external equipment; long-term nanomaterial safety unclear	Mainly preclinical; occasional large-animal evidence, but no established clinical application for post-tumour bone reconstruction
Antibacterial scaffolds	Prevention or suppression of infection through antibiotic release, antiseptic doping, antibacterial ions, or photothermal antibacterial activity	Antibiotic-loaded PMMA spacers, iodine-doped calcium phosphate cement, Zn/Cu-containing materials, LDH-based or photothermal antibacterial scaffolds [89,100–111]	Addresses a major complication of limb-salvage reconstruction; some platforms combine infection control with osteogenesis	Finite release duration; antibiotic resistance; possible ion- or nanoparticle-related cytotoxicity; inconsistent reporting of bacterial burden and infection recurrence	Antibiotic-loaded cement and spacers are clinically familiar; ion-doped and multifunctional antibacterial scaffolds remain largely preclinical
Angiogenic–osteogenic scaffolds	Coupled promotion of vascular ingrowth and new bone formation through growth factors, stem-cell-derived cues, bioactive ions, or surface modification	VEGF/BMP delivery systems, MSC- or BMAC/PRP-assisted scaffolds, copper/cobalt-doped materials, bioactive glass, modified HA scaffolds [117–148]	Supports vascularized bone regeneration; may improve repair of large defects; some biologic adjuncts are clinically accessible	Risk of ectopic ossification or aberrant vascularization; dose-dependent effects; uncertain influence on residual tumour cells; limited long-term oncologic safety data	BMAC, PRP, and selected bioactive materials have early clinical relevance; engineered growth-factor delivery systems remain mostly preclinical
Immunomodulatory scaffolds	Regulation of macrophage polarization, T-cell activity, cytokine signalling, or immune–osteogenic coupling to balance tumour control and regeneration	CSF-1R inhibitor-loaded scaffolds, cytokine-releasing hydrogels, checkpoint blockade delivery systems, HA- or graphene-HA-based immunomodulatory scaffolds [69,144–150]	Integrates immune control with tissue repair; may reduce recurrence while promoting regeneration	Complex and dynamic immune responses; risk of excessive inflammation or immune escape; simplified M1/M2 endpoints may not reflect true immune status	Early proof-of-concept stage; requires time-resolved immune profiling and long-term safety validation
Intelligent responsive delivery systems	Stimuli-responsive or staged release triggered by pH, enzymes, light, magnetic fields, oxidative stress, or programmed degradation	pH-responsive titanium scaffolds, MOF-based carriers, mesoporous silica/polymer systems, Janus hydrogels, sequential “kill-then-regenerate” platforms [43,50,76,77,155–170]	Enables spatiotemporal control; can match early tumour suppression with later regeneration; reduces nonspecific release	Patient-to-patient variability in tumour microenvironment; complex fabrication; unclear release reproducibility; lack of standardized paired tumour and repair endpoints	Predominantly preclinical; clinical translation depends on reproducible release kinetics and clinically relevant trigger validation
Gene-, cell-, or exosome-functionalized scaffolds	Delivery of nucleic acids, cells, extracellular vesicles, or biologic factors to modulate tumour behaviour, osteogenesis, angiogenesis, or immune response	Gene-activated matrices, siRNA-loaded nanoparticles, MSC-seeded scaffolds, exosome-loaded hydrogels or ceramics [51,68,125–135]	Strong biological regulatory potential; can target multiple regenerative and antitumour pathways	Stability, storage, biosafety, immunogenicity, tumour safety, and regulatory complexity; high manufacturing requirements	Early experimental stage; likely to face combination-product or biologic regulatory pathways
Additively manufactured patient-specific implants	Anatomical reconstruction with controlled macrostructure, porous architecture, and potential integration of drug delivery or bioactive modules	3D-printed titanium/tantalum prostheses, porous lattices, printed mesh scaffolds, hybrid metal–polymer–ceramic constructs [14,24,33,171]	Patient-specific fit; improved load transfer; controllable pore architecture; closer link to current reconstructive surgery	Cost; fatigue performance; quality control of porous structures; sterilization and batch consistency; limited long-term follow-up for multifunctional designs	Structural 3D-printed implants have entered clinical use; multifunctional or 4D printed bioactive constructs remain mostly preclinical

Overall, clinically familiar platforms such as antibiotic-loaded PMMA spacers, drug-eluting cements, bioactive ceramics, and patient-specific metallic implants appear closer to translation, whereas gene-activated, immune-modulating, 4D-printed, and multi-stimuli-responsive systems remain mainly proof-of-concept technologies requiring standardized models, long-term safety evaluation, and clearer regulatory pathways.

## Advanced manufacturing technology

Despite the promise of intelligent, microenvironment-responsive therapies, their reliability and clinical scalability depend on whether complex structure–function designs can be manufactured reproducibly with controlled quality. This section reviews macrostructural fabrication, microstructural tailoring, and interfacial/surface engineering, with emphasis on the process challenges that govern performance consistency, sterilization stability, and quality control.

### Macrostructural fabrication

Restoration of large skeletal defects after oncologic resection requires implants that replicate native anatomy, restore load-bearing function, and provide a stable biological interface. At this scale, macrostructure determines overall geometry and joint congruency, which are prerequisites for long-term function. While micro- and nanoscale modifications can refine biological performance, macrostructural fidelity remains the foundation for mechanical integrity and clinical stability.

Additive manufacturing has become the dominant strategy for producing customised implants. Patient-specific prostheses generated from CT or MRI data allow accurate matching of resection margins and joint surfaces [24,33]. Clinical series have shown that 3D-printed titanium or tantalum prostheses enable limb-sparing reconstruction with preserved joint motion and high patient satisfaction. The capacity to reproduce complex geometries directly impacts surgical precision and reduces intraoperative adjustments.

In periarticular sites, printed mesh scaffolds have been applied to restore subchondral support while preserving cartilage integrity. Early clinical use after curettage of benign tumours demonstrated functional recovery without implant-related complications [14]. Compared with solid spacers, mesh designs reduce stress shielding and promote bone ingrowth, making them suitable for sites requiring delicate load transfer.

Polymeric or composite constructs offer alternatives when metallic stiffness or infection risk is problematic. Antibiotic-loaded cement spacers can be shaped intraoperatively to fill irregular cavities, providing stability with local infection control, and follow-up studies report durable fixation at lower cost than modular metal implants [15,89].

An emerging advantage of macrostructural fabrication is its ability to integrate therapeutic functions. Examples include pH-sensitive titanium scaffolds releasing chemotherapeutics, bisphosphonate-loaded polymer frameworks, and photothermal ceramic composites, all of which combine structural support with tumour suppression [43,50,172]. These designs illustrate how macro-fit and biological activity can converge in a single device.

Clinical comparisons reinforce the value of precise macrostructural reproduction. In sacroiliac joint reconstructions, 3D-printed prostheses shortened operative time and improved functional scores compared with conventional cement-and-screw fixation, without increasing complication rates [171]. In the corresponding retrospective case-control study (12 vs 12; follow-up 6–90 months, median ∼21 vs 20 months), Musculoskeletal Tumor Society 93(MSTS-93) was evaluated at follow-up (24.1 ± 2.8 in the 3D-printed group vs 18.9 ± 2.6 in controls) and complication rates were similar between groups, supporting anatomical fidelity as both a mechanical and functional determinant [171]. Together, these findings suggest that high-fidelity anatomical restoration contributes meaningfully to long-term function.

### Microstructural tailoring

#### Design parameters

After tumour resection, scaffold microarchitecture is a primary design lever that co-determines mechanical competence, transport, and downstream bioactivity. Key parameters include pore size distribution, porosity/interconnectivity, unit-cell topology, strut/wall thickness, curvature, and intentional gradients/anisotropy, which jointly shape permeability and the local stress/strain field experienced by regenerating tissue [173–175].

#### Mechanical regulation

Architecture controls effective stiffness/strength and the spatial distribution of interfacial micromotion, thereby modulating stress shielding and mechanical stability. Triply periodic minimal surface (TPMS) designs provide a mathematically tractable route to tune stiffness–permeability trade-offs *via* curvature and connected pore networks [174,176]. Functionally graded/anisotropic lattices further allow direction-specific load transfer to match heterogeneous bone mechanics [177].

#### Metamaterial architectures as regulators of mechanics–biology coupling

Mechanical metamaterials (TPMS, auxetic lattices, topology-optimised lattices, and graded/hierarchical superstructures) translate geometry into coupled mechanical–fluidic fields that regulate cell fate. Auxetic (negative Poisson’s ratio) designs can alter deformation modes and strain transfer, potentially improving interlocking and mechanotransduction. Recent reviews summarize their biomedical/orthopaedic potential and AM routes [178,179]. Topology-optimised architected metals offer a framework to meet competing objectives (stiffness, fatigue, permeability) under AM constraints [180,181]. Notably, two-stage deformation metamaterial scaffolds have been reported to decouple strength from ultra-low effective modulus, increasing tissue strain while maintaining load capacity and enhancing osteogenesis/angiogenesis in critical defects [182].

##### Biological regulation

Microarchitecture regulates mass transport and solid mechanics, thereby influencing osteo-immune signalling, vascular ingrowth, and osteogenesis [180,183]. Within this architecture-defined boundary condition, surface and nanoscale functionalization can add therapeutic functions, including photothermal ablation, immune modulation, osteoconduction, and drug delivery [144,184–186].

##### Manufacturability & QC

Clinical translation hinges on reproducible microfeatures and preserved bioactivity. Because architected performance is defect-sensitive, micro-CT–based geometric quality control, fatigue-relevant testing, and transport validation are essential [187]. Scalability, sterilization-compatible bioactivity, batch consistency, and regulatory-aligned documentation remain key constraints, particularly for graded or multi-material designs [180]. Recommended reporting and QC endpoints are summarised in Table 1.

**Table 1.** Reporting framework for metamaterial-informed microstructural tailoring.

Architecture class	Design parameters	Mechanical endpoints	Biological endpoints	AM process	QC metrics
TPMS (Gyroid/Diamond/Primitive)	Porosity; minimal pore throat; curvature; wall thickness	E-modulus; yield/ultimate; fatigue; torsion	Bone ingrowth; BIC; BV/TV; vascular density	LPBF/EBM; DLP/DIW	micro-CT wall-thickness deviation; defect density; permeability (exp/CFD)
Auxetic lattices (NPR)	Unit cell; target Poisson’s ratio; anisotropy	Cyclic stiffness retention; interlock surrogate; fatigue	Mechanotransduction markers; integration strength	LPBF/EBM; VP	micro-CT strut deviation; anisotropy index; cyclic curve drift
Topology-optimized lattices	Objective/constraints; min strut; overhang	Stress shielding index; fatigue life	Infiltration depth; remodeling	LPBF/EBM	defect mapping; batch variance stats; permeability validation
Graded/anisotropic & hierarchical superstructures	Gradient function; multiscale units	Directional modulus; two-regime deformation	Spatially patterned ingrowth/angiogenesis	LPBF/EBM; hybrid	spatial micro-CT gradients; post-fatigue geometry retention

### Surface/interface modification & post-processing

As a post-processing (surface/interface modification) step rather than a primary manufacturing route, atomic-scale interface engineering tunes surface chemistry and nanostructure to improve host-bone integration—particularly relevant in oncologic reconstructions where bonding must withstand high loads in a compromised biological environment. Unlike bulk changes, nanoscale adjustments can modulate interfacial energy, reactivity, and signalling, thereby influencing osteointegration and healing.

Patient-specific 3D-printed scaffolds provide the foundation for such refinements. Once macro- and micro-architecture is tailored, element doping and nanoscale coatings can optimize protein adsorption, cell adhesion, and osteogenic differentiation by shifting surface charge, wettability, and topography [33]. Clinically, surface tailoring in ceramics or polymers may help delay premature degradation and sustain mechanical support until new bone forms. Interfaces can also be designed for sequential release of bioactive molecules, although release kinetics and retention after sterilisation are often not reported and should be verified with paired bioactivity and mechanical/QC readouts.

Key bottlenecks are reproducibility and robustness: sterilisation and cleaning can measurably alter surface chemistry and wettability, potentially changing protein adsorption and early cell responses. Process validation, therefore, requires geometry/feature fidelity checks (e.g. micro-CT for near-surface defects), surface characterisation (e.g. XPS, FTIR, contact angle, roughness), and durability testing after post-processing and sterilisation. These expectations align with regulatory emphasis on AM process controls and post-processing characterisation [188,189].

### Composite manufacturing techniques

Composite manufacturing aims to meet the dual clinical needs of stability and function after bone tumour resection. By combining polymers, ceramics, and therapeutic agents, scaffolds can restore load-bearing capacity while addressing residual disease and delayed healing.

Polymer–ceramic systems such as PCL/HA provide tunable degradation and osteoconductivity, supporting new bone formation while incorporating chemotherapeutics for local tumour suppression [189]. However, comparative reporting of paired endpoints—tumour burden/recurrence together with bone formation (micro-CT or histology)—is often not reported or varied across studies. Adding photothermal agents, including black phosphorus or copper-based nanoparticles, enables on-demand hyperthermia to ablate osteosarcoma cells while maintaining osteogenic activity [189]. Angiogenic composites (e.g. copper-modified bioceramics) further couple vascular induction with bone regeneration [189], ideally supported by co-reported vascular readouts (e.g. CD31 or vessel density) alongside bone indices.

Hydrogel–scaffold hybrids extend this concept by delivering growth factors like BMP-7 to accelerate angiogenesis and skeletal repair [190,191]. Other approaches, including semiconducting nanocrystals and doped bioactive glasses, have shown similar potential for integrating osteoinduction with tumour suppression [171,191,192], but head-to-head controls (free drug vs scaffold; trigger-on vs trigger-off) and standardized safety readouts remain inconsistent.

Key bottlenecks are process-driven: geometric fidelity and defect control (e.g. ceramic dispersion and pore interconnectivity), sterilisation/storage effects on mechanics and release behaviour, and batch-to-batch QC (micro-CT for architecture, mechanical testing, and release-kinetics verification). Representative guidance emphasizes documenting AM process controls and post-processing/characterisation for medical devices, while qualification principles for AM production sites highlight quality-relevant factors across operations. Sterilisation can also measurably alter properties of 3D-printed medical materials and should be validated for the specific composite system [193].

### Clinical rapid-response manufacturing

Rapid-response manufacturing provides timely, patient-specific implants or spacers after bone tumour resection by combining rapid imaging, digital modelling, and additive manufacturing. Its primary value lies in delivering immediate structural stability and local therapy in urgent situations.

A notable case involved an eight-year-old with deep infection following osteosarcoma resection. A CT-based, additively manufactured mould was used intraoperatively to cast an antibiotic-loaded PMMA spacer, ensuring anatomical fit and local drug delivery. The infection resolved, limb salvage was achieved, and partial joint recovery followed within three months [89]; longer-term durability endpoints were not reported.

Custom-printed titanium implants have also been used for complex pelvic reconstructions. Compared with cement-and-screw techniques, sacroiliac reconstructions with printed prostheses achieved shorter operative times and higher MSTS-93 functional scores, while maintaining similar complication and survival rates. Durable integration and mobility improvements were observed beyond 20 months of follow-up [171]; in a representative retrospective case-control cohort (12 vs 12; follow-up range 6–90 months), MSTS-93 was assessed at follow-up and complication rates were comparable.

Preoperative 3D-printed moulds for PMMA spacers further improve efficiency. In cases of infection, tumour, or necrosis, geometric conformity exceeded 90%, reducing surgical time and intraoperative adjustments. These spacers provided immediate defect filling, restored biomechanics, and supported early mobilisation without fracture or migration [194]. Study design, follow-up duration, and failure definitions were not reported here and should be specified for comparability.

Clinically, rapid-response manufacturing bridges the gap between acute defect management and definitive reconstruction. It supports early recovery, infection control, and preservation of limb function. Translation depends on controllable tolerances and defect management, sterilisation and storage stability, and batch-to-batch QC, including micro-CT for fit/porosity, mechanical testing, and, when drug-eluting systems are used, release-kinetics verification. These requirements align with regulatory expectations for AM process controls and post-processing characterisation [195] and with qualification principles for AM production sites [196].

## Clinical applicability and regulatory considerations

Clinical implementation should be guided by both biological efficacy and practical feasibility. Strategies based on clinically familiar platforms, including antibiotic-loaded PMMA spacers, drug-eluting cements, bioactive ceramics, and patient-specific metallic implants, may have a shorter translational path because their surgical handling, sterilization, and mechanical requirements are relatively well understood. By contrast, immune checkpoint-releasing scaffolds, gene-activated matrices, cell- or exosome-loaded constructs, and multi-stimuli-responsive nanocomposites require more extensive validation before clinical use.

Manufacturing standardization is a central barrier. For patient-specific implants, the workflow from imaging segmentation and computer-aided design to printing, post-processing, sterilization, and final quality control must be reproducible. For porous, degradable, or drug-loaded scaffolds, critical parameters include pore architecture, mechanical strength, fatigue resistance, degradation behaviour, drug-loading efficiency, and release kinetics. Sterilization may also alter polymer networks, hydrogel mechanics, protein activity, or drug-release profiles, making sterilization stability a necessary part of preclinical validation.

Regulatory pathways will depend on scaffold complexity. Purely structural implants may follow medical-device pathways, whereas drug-eluting, biologic-loaded, gene-activated, or immune-modulating scaffolds are more likely to be considered combination products. These systems require integrated evaluation of device performance, pharmacological activity, local and systemic toxicity, tumour safety, and long-term degradation. Cost-effectiveness is another practical issue: multifunctional or patient-specific implants must demonstrate measurable clinical benefits, such as fewer complications, lower revision rates, improved limb function, or better local disease control, to justify higher manufacturing costs.

## Conclusions

Postoperative reconstruction of bone tumours is shifting from passive structural replacement toward biofunctionalized therapy. Conventional substitutes restore stability but often fail to achieve durable integration or address the hostile tumour microenvironment, underscoring the need for new approaches. Recent advances show that scaffolds can integrate tumour suppression, infection control, immune regulation, and osteogenic–angiogenic stimulation, thereby addressing key limitations of traditional implants. However, most evidence remains preclinical and heterogeneous, and translation is restricted by model validity, endpoint inconsistency, regulatory hurdles, and production costs.

Advanced manufacturing enables patient-specific implants that replicate anatomy while embedding therapeutic functions. Hierarchical design spanning macrostructure through microarchitecture and interfacial engineering offers a pathway to synchronize stability with regeneration, although reproducibility and scalability remain significant barriers. At present, most multifunctional scaffolds should be regarded as promising but clinically immature platforms. Their future impact will depend on whether preclinical efficacy can be reproduced in standardized large-animal, load-bearing, tumour-relevant models and ultimately validated in prospective clinical studies with long-term follow-up. Ultimately, biofunctionalized multimodal implants have the potential to redefine limb-salvage surgery by uniting durable mechanical repair with effective local tumour control.

## Key messages

1. Biofunctional scaffolds are shifting bone tumour reconstruction from passive filling to active therapy, offering real potential to unite durable stability with local tumour control.

2. Clinical translation is closest with antibiotic-loaded cements and custom 3D-printed implants, which already support infection control and anatomical restoration in patients.

3. Future practice will depend on long-term safety validation and standardized manufacturing, but these multimodal implants hold promise to redefine limb-salvage surgery.

Compliance with Ethical Standards

## Data Availability

This review is based entirely on previously published studies that are available in peer-reviewed journals and public databases. No new data were generated or analyzed in this work.
